# Influence of Surface Preparation Method on the Bond Behavior of Externally Bonded CFRP Reinforcements in Concrete

**DOI:** 10.3390/ma12030414

**Published:** 2019-01-29

**Authors:** Sérgio Soares, José Sena-Cruz, José Ricardo Cruz, Pedro Fernandes

**Affiliations:** Institute for Sustainability and Innovation in Structural Engineering (ISISE), Department of Civil Engineering, University of Minho, Azurém Campus, 4800-058 Guimarães, Portugal; a65181@alunos.uminho.pt (S.S.); a51314@alunos.uminho.pt (J.R.C.); pmgfernandes@outlook.com (P.F.)

**Keywords:** EBR, CFRP laminates, concrete surface preparation, roughness characterization, bond behavior, analytical predictions

## Abstract

In last decades significant investigation has been carried out in order to predict the bond strength of externally bonded reinforcement (EBR) systems with carbon fiber reinforced polymer (CFRP) materials in concrete and, as consequence of that, many analytical expressions can be found in the literature, including in standards. However, these expressions do not account for the influence of several parameters on bond behavior such as the type of surface preparation which is a mandatory and critical task in the strengthening application. The present work gives contributions to reduce this lack of knowledge. For this purpose, an experimental program composed of single-lap shear tests was carried out, the main parameters studied being: (i) the type of concrete surface preparation (i.e., grinding and sandblasting) and (ii) the bond length. Prior to the application of the EBR CFRP system, the roughness level provided by the different methods of surface preparation was characterized by a laser sensor. Test results revealed that sandblasting concrete surface preparation yielded higher values, in terms of maximum shear force and fracture energy. Finally, existing expressions in standards were upgraded in order to account for the concrete surface roughness level in the estimation of the bond strength.

## 1. Introduction

In last decades, fiber reinforced polymer (FRP) materials have been increasingly proposed to strengthen reinforced concrete (RC) structures [1,2,3]. This approach has been demonstrated as an effective alternative solution to the traditional strengthening methods which typically employ reinforced concrete and/or steel. FRP materials have shown to be a useful and efficient solution due to their good mechanical properties, lightness, resistance to corrosion, low thermal conductivity, good fatigue behavior and easy installation procedures [4,5]. Different strengthening techniques have been developed for applying FRP. The first one, Externally Bonded Reinforcement (EBR), appeared in the 1980s [6,7], where the FRP laminate is glued onto the tensile face of the RC element. A few years later, other techniques emerged such as the near-surface mounted (NSM), where the FRP laminate or bar is inserted onto a groove previously cut in the concrete cover. According to the literature, when FRP are used as reinforcement material, bond behavior between this and concrete substrate is generally a critical issue for both EBR [8,9,10,11,12,13,14,15,16,17,18,19] and NSM techniques [13,16,20,21,22,23,24] that directly influence the effectiveness of the structural reinforcement. The strengthening performance of these techniques depends significantly on the resistance of the concrete cover, which is normally the most degraded concrete region in the structure and the concrete surface condition. As a result, premature failure of FRP reinforcement can occur and, generally, its full mechanical capacity is not mobilized, mainly when adopting the EBR technique. In attempt to overcome these issues, other strengthening techniques with composite materials have emerged such as Mechanically Fastened and Externally Bonded Reinforcement (MF-EBR) [4,25], which uses multi-directional carbon fiber laminates, simultaneously glued and anchored to concrete, and Mechanically Fastened FRP (MF-FRP) [25,26,27,28,29] using only steel anchors to fix the laminate to the substrate. Despite their potential, these techniques also reveal some shortcomings such as (i) the possible concrete damage during anchoring, (ii) the limited opportunity of anchor’s installation in the presence of congested internal reinforcement in the members to be strengthened and (iii) other problems related to the stress concentration and bearing failures of the systems leading to slippage of FRP and loss of bond.

The present work focuses on the bond behavior characterization between EBR CFRP system reinforcement and the concrete surface. As many researchers verified in their experimental works when this technique was applied, the bond strength depends on several factors, including mechanical and physical properties of concrete, composite material and epoxy adhesive which prevent the fully FRP tensile strength to be attained due to the premature debonding failure that typically occurs [12,13,15]. Moreover, it is well known that the concrete surface preparation and bonding technique are crucial tasks for adequate installation of an EBR FRP system to delay the brittle debonding failure. Therefore, it is a key issue to proceed a preparation method that ensures the cleanliness, the soundness and a proper concrete surface roughness level [11,12]. More specifically, the concrete surface preparation process includes the removal of unsound concrete, strength verification and opening of the pore structure. The methods that have been most used by the scientific community are sandblasting, grinding, brushing, scarifying, steel shot blasting and bush hammering with different advantages and disadvantages associated, in terms of roughness level provided, cost, processing time and application difficulties. Through bond characterization tests between concrete surface and FRP, some authors demonstrated that the bond strength is higher when an effective surface preparation method is used due to the higher surface roughness level that provides better adhesion along the system interface [11,12,18,19].

In terms of standard guidelines, the influence of the concrete surface roughness on the EBR system performance and bond strength has been recognized in a comprehensive manner [1,2,30,31]. According to the International Federation for Structural Concrete (fib) [2], the concrete substrate should be roughened and made laitance and contamination free, in such a way that the concrete quality can be utilized in an optimum way. This guideline recommends that the surface preparation is done preferably by means of high-pressure blasting (sand, grit, water jet blasting) or grinding. In turn, the ACI Committee 440 [31] proposes that at least the application of abrasive or water blasting techniques for surface preparation should be performed. On the other hand, the Italian National Research Council [1] highlights the importance of a quality control that includes the determination of concrete conditions, removal of any deteriorated or loose concrete, cleaning and protection from corrosion of existing steel reinforcement, and finally substrate preparation for receiving the selected FRP reinforcement. In accordance with this standard, once the quality control of the substrate had been performed, the deteriorated concrete had been removed, the concrete cross section had been restored, and the existing steel reinforcement had been properly treated, sandblasting of the concrete surface shall be performed.

Although the main guidelines recognize that concrete surface preparation prior the installation of the EBR FRP is an important task, the analytical formulations provided to predict the system bond strength still do not consider the influence of the use of different methodologies to do that. Possible reasons for this reside on the fact that different concrete preparation methods provide different roughness levels of the surface. Additionally, sometimes it is difficult to obtain a uniform surface roughness level, because this depends on human skills in the execution of the surface preparation method and it becomes a critical aspect when specimens under the same test conditions are produced. Therefore, a good scientific development would be to quantify the roughness level of the concrete surface before the reinforcement procedure and to understand how this influences the bond behavior of the EBR FRP system, when different methodologies of surface preparation are used. Then, the next step would be to introduce this influence on the bond strength prediction formulations. To measure the concrete surface roughness level after the application of different methods of surface preparation, several authors have used lasers profilometers [11,32,33]. This approach has proven to be very effective for quantifying the roughness level of the concrete surface, and can be the key to correlating this result with the bond behavior of the strengthening system. The use of this technology is something that is feasible in fieldwork, namely in a real application of the EBR FRP system. Nowadays, there are already commercial and patented systems that allow the assessment of the quality control of the concrete surface preparation. Thus, concrete surface roughness should be a requirement at the execution stage of the intervention and subject to quality control before proceeding to the concrete element strengthening.

The present paper aims at contributing to a better understanding the influence of the use of different concrete surface preparation methodologies on the bond behavior of CFRP EBR systems. For this purpose, an experimental program composed of single-lap shear tests was carried out. This was performed by adopting an experimental set-up developed by the authors and the main parameters studied were the type of concrete surface preparation (SB—sandblasting and GR—grinding) and the bond length between CFRP and concrete surface. Prior to the application of the EBR CFRP system, the roughness level provided by the different methods of surface preparation was characterized by a laser sensor, in order to make it possible to correlate these measurements with the results obtained in the bond tests which characterize the interface behavior between concrete and CFRP laminate. Then, the formulation proposed by the Italian National Research Council [1] was updated in order to account for the roughness level of the concrete surface on the estimation of the bond strength.

## 2. Experimental Program

### 2.1. Test Program

The main objective of the present investigation was to characterize the bond behavior of concrete elements with EBR CFRP systems, considering the following study variables: (i) the concrete surface preparation method and (ii) the bond length. The program was composed of 24 single-lap shear tests divided into two main groups according to the concrete surface preparation methodology used: (i) GR—grinding and (ii) SB—sandblasting. The selection of these two methods of concrete surface preparation was based on the fact that these two have been the most used in structural strengthening with the EBR technique. In each group, three different bond lengths (L_b_) were considered: (i) 150 mm, (ii) 200 mm and (iii) 250 mm. According to some provided analytical formulations [1,2,8], the theoretical value of the effective bond length ranges between 191 and 217 mm. This means that the L_b_ values adopted represent, respectively, a lower, an approximate, and a higher value than the theoretical effective bond length. Thus, it is possible to perform an analysis of the influence of the L_b_ adopted on the EBR CFRP system bond behavior. Consequently, six series were adopted, each one composed of four specimens in the same conditions in order to ensure reliable results. The generic denomination of the test specimens is X_LbY_Z, where X represents the concrete surface preparation method (GR or SB), Y is the bond length in millimetres (150, 200 or 250) and Z is the specimen order number tested under the same conditions (1, 2, 3 or 4).

### 2.2. Specimen’s Geometry, Experimental Set-Up and Instrumentation

**Figure 1:**
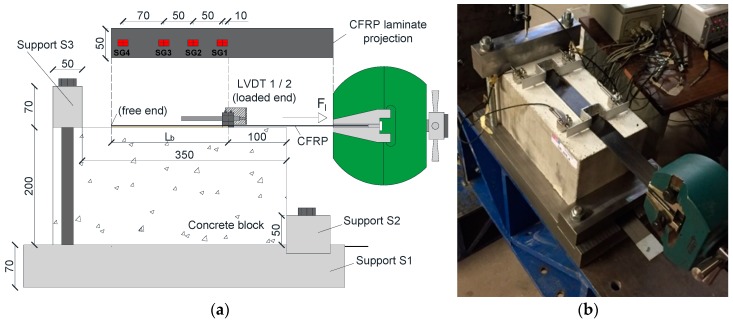
Experimental set-up and instrumentation: (**a**) scheme; (**b**) photo. Note: units in [mm].

Figure 1 depicts the test set-up and instrumentation adopted in the experimental program for the case the specimen with a L_b_ of 200 mm. Concrete blocks of 400 × 200 × 200 mm^3^ were used, where two CFRP laminate strips were applied in opposite faces (parallel to the casting direction), according to the EBR technique. Pultruded CFRP laminate strips with 50 mm of width and 1.2 mm of thickness were used. To avoid premature failure by concrete rip off ahead of the loaded end, the bonded lengths started 100 mm apart, from the extremity. The concrete specimen was placed horizontally on a steel plate (support S1) with 70, 300 and 550 mm of thickness, width and length, respectively. Support S1 was fixed to the stiff base of the testing steel frame system through eight M16 steel threaded rods. To assure negligible horizontal displacements of the concrete block in the loading direction during the test, a steel plate (support S2) was placed in the bottom front part of the concrete block, which acted as a reaction element at a height of 50 mm. To minimize vertical displacements during the test, a steel plate (support S3) of 50 × 70 × 300 mm^3^ was placed in the rear top part of the concrete block. Both support S2 and S3 were fixed to the support S1 through two M20 steel threaded rods. The tests were performed using a servo-controlled equipment (INEGI, Porto, Portugal). The applied force was measured through a load cell of 200 kN maximum carrying capacity (linearity error of 0.05% F.S.), placed between the actuator and the grip used to fix the CFRP laminate during the test. The relative displacement between the CFRP and the concrete (slip) at the loaded end section (*s_l_*) was computed as the average of displacements measured by the linear variable displacement transducers (LVDT) 1 and 2 with a stroke of ±5 mm (linearity error of 0.24% F.S.). The tests were performed under displacement control at the loaded end through LVDT2 (rate of 2 μm/s). Additionally, a series of four strain gauges TML BFLA-5-3-3L (SG1 to SG4) were placed along the CFRP laminate centerline to measure the longitudinal strains during the loading process (Figure 1).

### 2.3. Specimen’s Preparation

**Figure 2:**
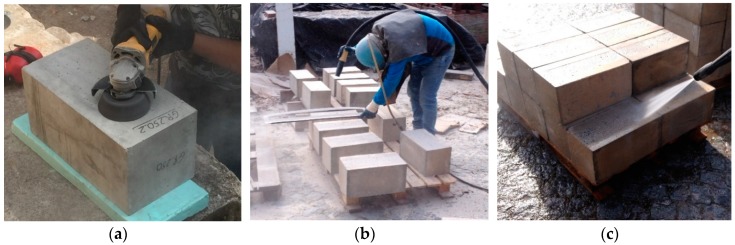
Concrete surface preparation: (**a**) grinding; (**b**) sandblasting; (**c**) surface cleaning.

According to the test program, two different methods of concrete surface preparation were studied. The surface preparation consists of removing the superficial concrete layer with inappropriate characteristics in order to obtain a clean and healthy surface with absence of the laitance layer and with an adequate roughness level, showing the aggregates. The aim was to understand the influence of the different methodologies in terms of providing concrete surface roughness and its consequence on the bond behavior of the EBR system. The two concrete surface preparation methods used in the experimental program are presented in Figure 2: (i) GR—grinding and (ii) SB—sandblasting. Both surface preparation methods used are relatively easy to apply. The GR is a mechanical method of surface preparation and consists of removing the unsound concrete layer through rotating shafts with sprockets that progressively wear out the surface layer. In this case, a grinding wheel was used until the aggregates had become visible (see Figure 2a). In turn, SB is a process of surface preparation based on particles’ impacts, being one of the most used techniques. This consists of the projection of small particles against the surface in order to cause surface abrasion and wear due to the high speed of projection (see Figure 2b), allowing a faster concrete surface preparation than the GR method. However, the SB method application requires professional equipment and skilled labor. After surface preparation, the treated surface was cleaned by high-pressure water jet (Figure 2c) to remove the debris and dust resulting from the treatment applied, ensuring a good adhesion between the concrete surface and the reinforcing system. The next steps of preparing the specimens were performed after the specimens became dry.

### 2.4. Concrete Surface Roughness Characterization

**Figure 3:**
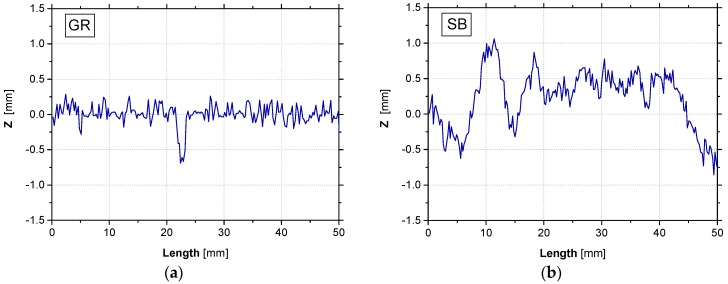
Roughness profile when different surface preparation methods were applied: (**a**) grinding; (**b**) sandblasting.

Considering the different preparation methodologies of the concrete surface (GR and SB), the roughness of these distinct surfaces was measured and analyzed. The main purpose was to relate the different methodologies to the provided concrete surface roughness and with the bond between CFRP laminate and concrete substrate. The procedure involved the use of a laser sensor, whose main characteristics are presented in Table 1. Three longitudinal profiles (with 20 mm apart from each other—transverse direction) were analyzed by the laser sensor in each of the 24 surface areas where the EBR CFRP system was installed. The roughness measurement through the laser sensor allowed to obtain qualitative and quantitative information of the different surfaces. The configuration of the surface roughness measuring system is also stated in Table 1. The laser sensor was connected to a metallic plate, which was part of a mechanism conceived to produce a slow displacement at a constant rate, as well as to allow the displacement of the laser sensor during scanning at a parallel and rectilinear path relatively to the scanned surface. The displacement rate was 8.8 mm/s and the data acquisition rate was 34 Hz, leading to consecutive readings spaced at 0.3 mm. Representative roughness profiles provided by the two methods of surface preparation and obtained by the laser sensor along 50 mm of the bonded length are shown in Figure 3. The results clearly show that the SB method produced a higher level of surface roughness than the GR method.

To quantitatively characterize the roughness level provided by each of the surface preparation methods several statistical indicators were considered, such as the mean roughness coefficient (R_m_) and the root mean square (R_q_). These indicators can be determined using Equations (1) and (3), respectively. In these equations, the parameters *l*, *z*(*x*) and *n* are the evaluation length, the profile height at position *x* and the number of scan readings performed by the laser sensor, respectively.

(1) $$R_{m}=\frac{1}{l}\;\int_{0}^{l}\left|z\left(x\right)-\bar{z}\right|\cdot dx\;\approx \;\frac{1}{n}\;\overset{n}{\underset{i=1}{\sum }}\left|z_{i}-\bar{z}\right|,$$

(2) $$\bar{z}=\frac{1}{l}\;\int_{0}^{l}z\left(x\right)\cdot dx\;\approx \;\frac{1}{n}\;\overset{n}{\underset{i=1}{\sum }}z\left(x\right),$$

(3) $$R_{q}=\sqrt{\frac{1}{n}\;\overset{n}{\underset{i=1}{\sum }}z_{i}^{2}}.$$

**Figure 4:**
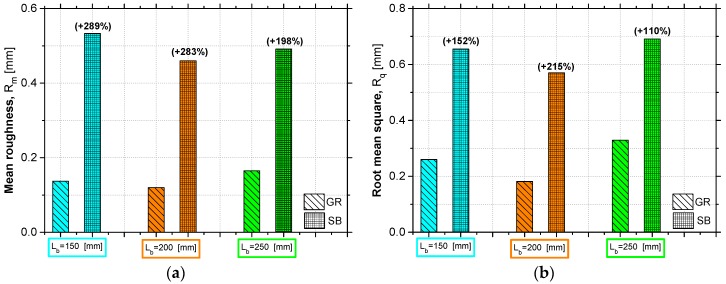
Roughness level comparison provided by each surface preparation method (GR and SB) in terms of: (**a**) mean roughness (R_m_); (**b**) root mean square (R_q_). Note: the values in parentheses represents the percentage increase when SB was applied instead of GR.

The statistical indicators used were then computed for the two different types of surface preparation and the results from the roughness assessment are presented in Table 2. The comparison between the value of the parameters R_m_ and R_q_ provided by each surface preparation method is established in the Figure 4.

The results of each statistical indicator were obtained by the average value of the four test specimens with the same test conditions, i.e., the specimens with the same bond length and the same concrete surface type. According to the results, it is possible to verify that the SB method allowed to obtain considerably higher roughness levels than the GR. In terms of R_m_ and R_q_, the SB method allowed an average increase of 250% and 150%, respectively, in relation to the surface preparation with GR. It is important to note that the values of coefficient of variation (CoV) are sometimes significant due to the difficulty of obtaining a uniform surface roughness level. This depends on the human skill in the execution of the surface preparation method, as well as on the homogeneity of the material, and becomes a critical aspect when specimens under the same test conditions are produced, in which, theoretically, the same levels of roughness would be desired.

### 2.5. Material Characterization

The material characterization included the assessment of the mechanical properties of the materials involved in this experimental program, mainly: (i) concrete, (ii) CFRP laminate strip and (iii) epoxy adhesive. Table 3 shows the obtained results.

Only one batch was used for casting all the test specimens. The used concrete had the following characteristics: class C25/30; exposure class XC2; maximum aggregate size of 12.5 mm; slump S4. The average Young’s modulus (*E*_cm_) and average compressive strength of concrete (*f*_cm_) were assessed using five cylinders with 300 mm of height and 150 mm of diameter and following the recommendations NP EN 12390-13:2014 [34] and NP EN 12390-3:2011 [35], respectively, at 28 days of concrete age.

The CFRP laminate strips used in the experimental work (type: S&P Laminates CFK) consists of unidirectional carbon fibers held together by an epoxy vinyl ester resin matrix. These were produced by S&P^®^ Clever and Reinforcement with the trademark CFK 150/2000. According to the manufacturer, this type of CFRP laminate presents a smooth external surface and the fiber volume content is about 70%. Pultruded CFRP laminate strips with 50 mm of width and 1.2 mm of thickness were used. The tensile mechanical properties included the average Young’s modulus (*E*_f_), the average tensile strength (*f*_fu_) and the average strain at the peak stress (*ε*_fu_) were assessed using five samples and following the recommendation ISO 527-5:2009 [36].

The epoxy adhesive (type: S&P Resin 220 epoxy adhesive), produced by the same supplier as for the CFRP laminate, was used as bond agent to fix the reinforcing material to the concrete substrate. This adhesive is available in the form of two components (Component A—resin and Component B—hardener) that need to be mixed. According to the manufacturer, the ratio A:B in the mix should be 4:1. In the scope of the present experimental work, the epoxy adhesive was not characterized, and its mechanical properties were collected from a previous work performed by some of the authors of the present work [21]. In this context, six samples were prepared and tested following the recommendation ISO 527-2:2012 [37]. These results are also presented in Table 3, in terms of average tensile strength (*f*_a_), average Young’s modulus (*E*_a_) and average strain at the peak stress (*ε*_a_).

## 3. Results and Discussion

### 3.1. Main Results

In this section the main results obtained in the single-lap shear tests are presented. Table 4 summarizes the average results for each series obtained in the experimental program through several parameters that characterize the bond behavior between the CFRP and the concrete, mainly: *F_l_*_,max_ is the maximum shear force; *s_l_*_,max_ is the loaded end slip at *F_l_*_,max_; *G*_f_ is the total energy up to 0.3 mm of loaded end slip (common *s_l_* value attained in all the tests); *ε*_fmax_ is the CFRP normal strain at *F_l_*_,max_; and, finally, FM represents the failure mode observed in each specimen tested.

From a general analysis, a significant influence of the surface preparation method used on the bond behavior is verified. In fact, the SB method proved to be more efficient than GR because it allowed an increase in the bond strength against debonding failure. Therefore, when the same bond length was adopted, the SB method provided an increase of *F_l_*_,max_, *s_l_*_,max_, and *G*_f_ in relation to the GR method. In turn, the L_b_ adopted also showed to have influence on the bond behavior. With the increase of L_b_, a bond strength increase was observed, translated by the increase of the necessary shear force to induce debonding failure of the EBR system. Furthermore, when the same surface preparation method was used, the increase of L_b_ provided an increase of *s_l_*_,max_ and *G*_f_. In all tests, the debonding of the CFRP laminates occurred with a thin layer of concrete.

**Figure 5:**
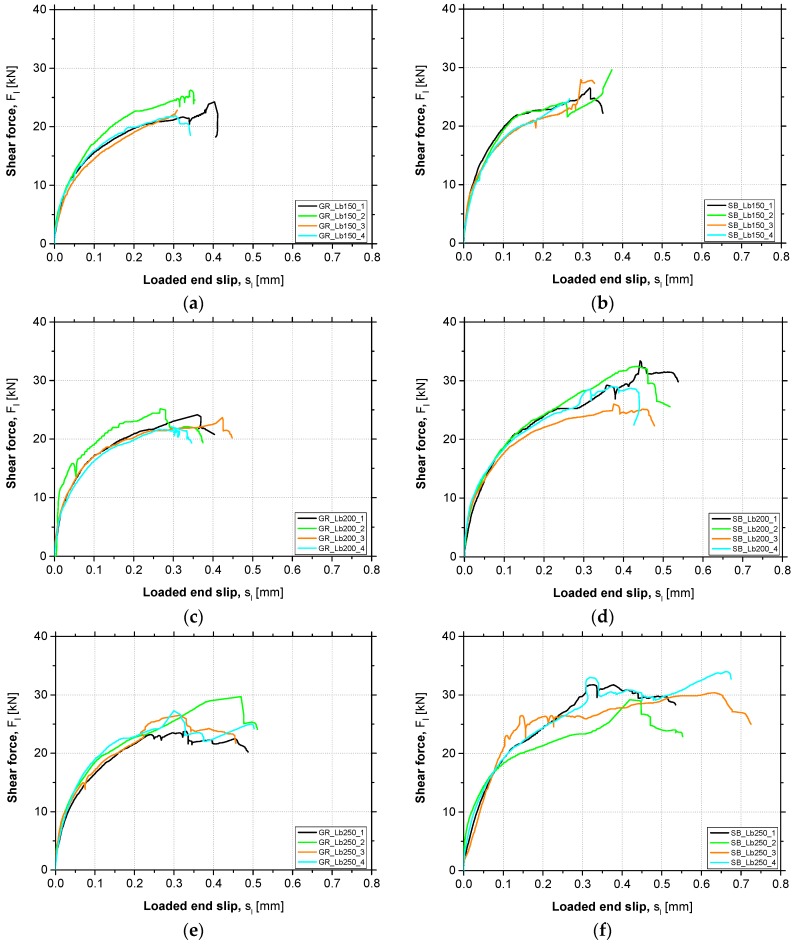
Shear force vs. loaded end slip obtained in each series: (**a**) GR_Lb150; (**b**) SB_Lb150; (**c**) GR_Lb200; (**d**) SB_Lb200; (**e**) GR_Lb250; (**f**) SB_Lb250.

The shear force vs. loaded end slip curves (*F_l_*-*s_l_*) obtained in all tests carried out in this experimental work are presented in Figure 5. In each graph, the responses corresponding to the four specimens tested under the same conditions are gathered. Later, a more detailed analysis about the influence of the study variables is performed.

In each *F_l_*-*s_l_* presented in the Figure 5, the three distinct phases in the behavior are observed regardless of the surface preparation method used and L_b_ adopted. The initial branch is almost linear, due to the behavior of the involved materials and perfect bond. After this phase, an increasing stiffness degradation can be observed due to the start of EBR CFRP bond degradation conditions. Then, an almost steady state phase can be observed, due to the debonding process. This process of failure starts when the ultimate shear strength is attained at the beginning of the bonded length, i.e., at the loaded end. During debonding failure, an increase in the loaded end slip at an almost constant value of applied shear force is observed. However, in some particular cases, after the initiation of the debonding process, a slight increase in the applied force was observed. This fact can be justified by possible heterogeneities in the concrete specimens, namely when the concrete layer, where failure occurs, crosses a coarse aggregate. This implies that a higher shear force would be required to increase the slip (friction at interface level). According to the responses presented in Figure 5, it is also important to highlight that the adopted test methodology proved to be adequate in providing a stable debonding process. Similar responses in terms of *F_l_*-*s_l_* curves have been obtained in other research studies, e.g., [12] or [13].

### 3.2. Failure Modes

As shown in Table 4, cohesive debonding in the concrete failure mode was observed in all the single-lap shear tests carried out, regardless of the surface preparation method used and bond length adopted. After debonding failure, a concrete layer attached to the epoxy adhesive and CFRP laminate was analyzed. This failure mechanism was also verified in others investigation works, e.g., [11,12].

During the loading process, inclined microcracks arose on the external concrete layer since the concrete tensile strength is lower than the corresponding adhesive’s strength. The emergence of these microcracks occurred from the loaded end to the free end section, accompanying the debonding process of the EBR CFRP system that starts when the bond strength is reached. Therefore, the bond strength is gradually lost in the successive loaded end sections during the loading process, and then other zones of bonded length are activated, providing its contribution to the stress transfer. While the debonding failure occurs, the stress distribution capacity in the successive loaded end sections become nil. The characteristics of the failure mechanism observed highlight the importance of the adhesive properties and the use of an effective surface preparation prior the installation of the EBR CFRP system that promotes a good adhesion between the concrete surface and the reinforcing material. These are crucial aspects in order to make it possible to increase the bond strength against debonding failure. Therefore, based on the analysis of the failure modes that occurred in the tests, important conclusions regarding the influence of the concrete surface preparation method used can be drawn.

**Figure 6:**
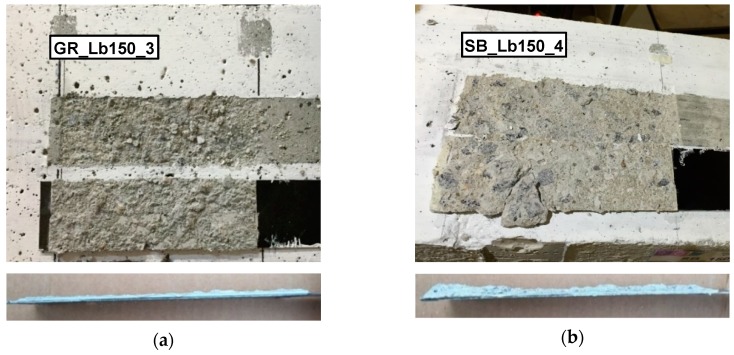
Fracture surface of carbon fiber reinforced polymer (CFRP)/concrete after debonding for different surface preparation methods: (**a**) grinding; (**b**) sandblasting.

Figure 6 illustrates two typical interface fracture surfaces for the case of GR and SB of concrete surface preparation. From a qualitative viewpoint, the effectiveness of the surface preparation methods used can be assessed by a visual analysis of the fracture surfaces. In the specimens where SB was used, a thicker concrete layer attached to the CFRP laminate after bond failure was found (Figure 6b). In contrast, a smaller amount of concrete attached to the CFRP surface was observed after bond failure in GR specimens (Figure 6a). In some cases, the concrete layer attached to the CFRP laminate was observed only in part of the bond length, being the remaining with neither concrete nor adhesive. This situation occurred mainly in the SB specimens with larger bond length (SB_Lb250_2, SB_Lb250_3 and SB_Lb250_4) and the reason may be related to the fact that this L_b_ value is higher than the theoretical effective bond length in stress transfer, promoting higher possibilities of having the influence of the existing heterogeneities in the concrete interfering with the bond conditions.

**Figure 7:**
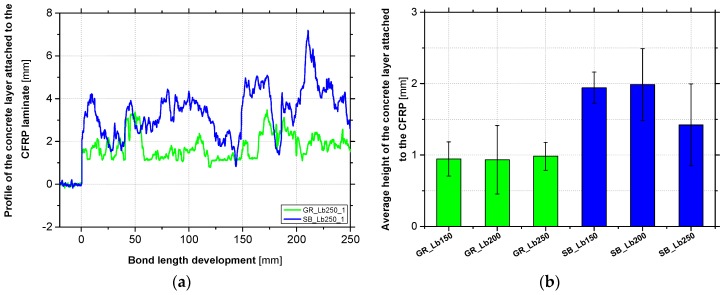
Concrete attached to the CFRP after debonding failure for the series Lb250 (**a**) and the average height of the attached concrete layer (**b**).

Additionally, from a quantitative viewpoint, the amount of concrete attached to the CFRP after debonding failure was analyzed using a laser sensor SICK OD2-P50W10A0 with the same characteristics as the laser sensor used in the assessment of the concrete surface roughness (see Table 1 in Section 2.4). The attached layer of concrete to the CFRP laminates was measured at the middle axis along the entire bond length. In these readings, the adopted displacement rate was 0.69 mm/s and the data acquisition rate was 120 Hz, leading to consecutive readings spaced at 0.0055 mm. Figure 7a compares two representative longitudinal profiles measured by the laser sensor along the bond length of 250 mm for the case of GR and SB methods. In order to have a measurement reference, a non-adhered zone of the CFRP just before the start of the bond length (without any concrete attached) was measured, and also presented in these figures. In turn, Figure 7b shows the results in terms of average height of concrete layer attached to the CFRP, considering a constant epoxy adhesive thickness of 1 mm along the bond area. In this latter figure, the standard deviation obtained in each series is also included.

These results highlight that when SB was used, a thicker concrete layer attached to the CFRP was obtained, as had been verified in previous visual analyses of the fracture surfaces. On average, a concrete layer with a height of 1 and 1.8 mm appeared attached to the CFRP when GR and SB methods were used, respectively. As would be expected after the visual analysis of the fracture surfaces, a higher dispersion of the average concrete layer height was observed in the CFRP laminates of the SB specimens. In fact, this surface preparation method proved to be more effective to the bond behavior and made it possible to pull some coarse aggregates out of the concrete surface. In contrast, a weaker adhesion between the concrete and the reinforcing material was verified in GR specimens.

### 3.3. Influence of Studied Parameters on the Bond Behavior

In this section, a more detailed analysis of the influence of the study variables on the bond behavior between concrete and EBR CFRP system is carried out. Thus, the influence of concrete surface preparation methods used (GR and SB) and the bond length adopted (150, 200 and 250 mm) on the following key aspects is investigated: (i) response in terms of pull shear force vs. loaded end slip curves (*F_l_*-*s_l_*), (ii) maximum shear force (*F_l_*_,max_) and (iii) total energy (*G*_f_).

#### 3.3.1. Pull Shear Force vs. Load End Slip Curves (*F_l_*-*s_l_*)

**Figure 8:**
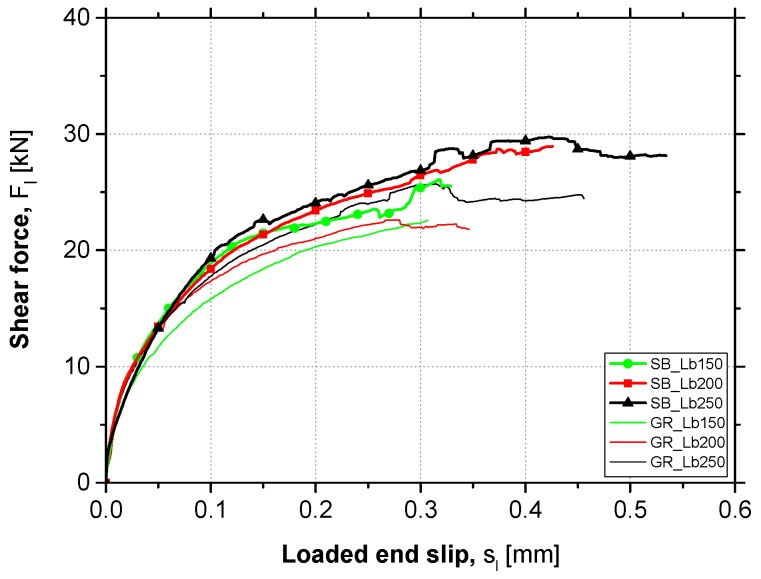
Average curves of the pull shear force vs. loaded end slip.

Figure 8 presents the average *F_l_*-*s_l_* curves obtained in each test series. Each curve was obtained through the mean results of the four tests carried out under the same conditions. By the analysis of the *F_l_*-*s_l_* curves presented, it can be observed that the preparation method of concrete surface used did not introduce considerable changes in the strengthening system initial stiffness, since the initial branch of the curves has a practically coincident slope. However, as the debonding process of the EBR CFRP system started, higher shear force values in the case of SB specimens compared to the GR specimens were observed. Therefore, it can be concluded that the SB method proved to be more effective than GR in terms of bond strength against debonding failure.

Thus, in turn, the bond length adopted did not influence the *F_l_*-*s_l_* curves shape neither in the linear initial branch nor in the stiffness degradation phase. Therefore, as can be seen in Figure 8 when comparing the curves with the same surface preparation method, the debonding failure process starts at very close shear force values, regardless of the L_b_ adopted. Nevertheless, higher values of maximum shear force and slip at the loaded end were obtained when higher L_b_ was used.

#### 3.3.2. Maximum Shear Force (*F_l_*_,max_)

**Figure 9:**
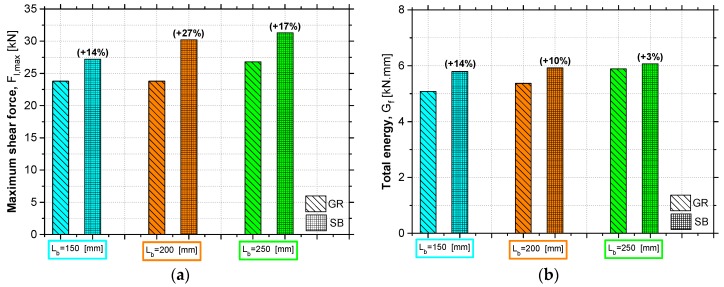
Influence of study parameters on the: (**a**) maximum pull shear force; (**b**) total energy. Note: the values in parentheses represents the percentage increase when SB was applied instead of GR.

Figure 9a shows the results obtained in terms of maximum shear force and allows to understand the influence of the concrete surface preparation method used and the bond length adopted. As expected, the SB method provided a higher bond strength between the concrete surface and the reinforcement system, with respect to the GR method. Thus, the influence of the roughness level presented by the concrete surface on the reinforcement efficiency according to the EBR technique became evident. In a more precise manner, when SB was used instead of the GR, an increase in *F_l_*_,max_ of 14%, 27% and 17% was obtained on the specimens with an L_b_ of 150, 200 and 250 mm, respectively. In fact, the specimens with L_b_ of 200 mm showed a more marked increase in *F_l_*_,max_ compared to the remaining test specimens. This can be explained by the fact that, in addition to the GR method being less effective than SB, the roughness level of the specimens with L_b_ of 200 mm was lower than the counterparts of 150 and 250 mm, as demonstrated by the lower values of R_m_ (see Table 2). It is also important to mention that since the concrete roughness level presents some variations, even when the same surface preparation method has been used, the influence of the bond length in the bond behavior is less significant because although it was expected to obtain higher maximum shear forces with the increase of L_b_, there is inherent results variation related to the concrete surface roughness which influence the effective bond length and the corresponding maximum shear force.

Nevertheless, in relation to the influence of the adopted bond length, it is possible to identify an increase in the *F_l_*_,max_ when the L_b_ value increases, but not in a proportional manner. This increase was more pronounced when the L_b_ value was changed from 150 to 200 mm, with respect to the change in L_b_ value from 200 to 250 mm, except for the GR specimens for the reasons explained above. Therefore, when the SB method was used, *F_l_*_,max_ increases of 11% and 4% were obtained when the L_b_ value was changed from 150 to 200 mm and from 200 to 250 mm, respectively. These results seem to suggest that the effective anchorage length, from which an increase of L_b_ does not provide an increase of *F_l_*_,max_, is between 200 and 250 mm, as will be proven analytically in the next sections.

#### 3.3.3. Total Energy (*G*_f_)

Figure 9b shows the results obtained in terms of total energy throughout the loading process until the moment that a slip of 0.3 mm was registered on the loaded end section. The analysis of these results also allows conclusions to be drawn about the effectiveness of the concrete surface preparation method used, as well as to notice the influence of the bond length adopted. According to the results, when SB was applied, the total energy dissipated until a *s_l_* value of 0.3 mm has been registered was 14%, 10% and 3% higher than that found in the GR specimens with 150, 200 and 250 mm of L_b_, respectively. Therefore, once again, the superiority of SB method in terms of reinforcement effectiveness was demonstrated when compared to the GR. This superiority would also be expected after analysis of the interface fracture surfaces (Figure 6) in which a thicker concrete layer attached to the CFRP laminate after bond failure was found in the SB specimens. This reflects the better bond behavior between CFRP and the concrete surface and justifies the higher values of *G*_f_ obtained.

In terms of the adopted bond length influence on the *G*_f_ values, increases of 6% and 10% were verified in the GR specimens when the L_b_ value was changed from 150 to 200 mm and from 200 to 250 mm, respectively. On the other hand, increases of 2% and 3% were observed on the SB specimens when the L_b_ value was changed from 150 to 200 mm and from 200 to 250 mm, respectively. In fact, the L_b_ value was shown to be less influential in the SB specimens, which may be justified by the fact that the total energy up to the proposed slip value is more dependent on the surface preparation method used and its effectiveness. In addition, and as mentioned in the above section, since there were inherent surface roughness variations even when the same surface preparation method was applied, the influence of the L_b_ value may be less significant with respect to the influence of the concrete surface roughness.

### 3.4. Interfacial Behavior

A series of four strain gauges (SG1 to SG4) were placed along the CFRP laminate centerline to measure longitudinal strains during the loading process in the specimens with L_b_ of 200 mm. The analysis of longitudinal strain distribution on the CFRP surface along the bonded length allows to understand the bond behavior and debonding failure process of the strengthening system during the test.

**Figure 10:**
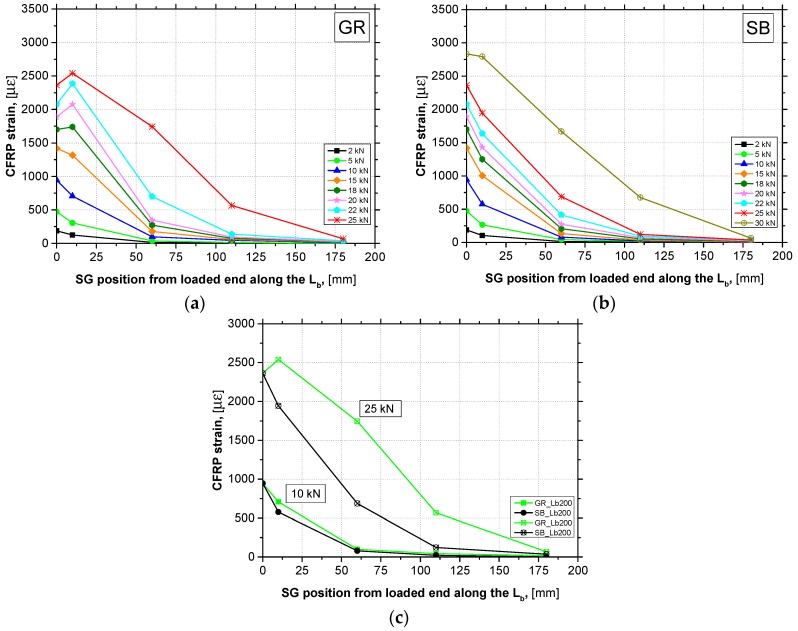
Longitudinal strains at different loading levels along the CFRP: (**a**) GR_Lb200; (**b**) SB_Lb200; (**c**) GR_Lb200 vs. SB_Lb200.

Figure 10a,b shows the CFRP longitudinal strain distribution, at different loading levels, from the loaded end to the free end side for a bonded length of 200 mm obtained in the GR and SB specimens, respectively. The first point of each of the curves plotted (strain at loaded end section—*ε*_0_) was analytically calculated by the following expression: *ε*_0_ = *F_l_*/(*E*_f_ × *A*_f_), where *F_l_*, *E*_f_ and *A*_f_ represent the force applied, the CFRP Young’s modulus and the CFRP cross-section area, respectively. The interface behavior presented by the GR and SB specimens was very similar due to the same failure mode occurring. Before the start of debonding process, an exponential decrease of the longitudinal strain from the loaded end section to the strain gauge installed near to the free end section (strain value close to zero) is remarkable. As soon as the debonding process starts, the longitudinal strains recorded by the SG1 (strain gauge closer to the loaded end) approximate of the strain values analytically calculated at the loaded end section, which means that the shear stresses are no longer transmitted at the interface level in this section. At this phase, the typical S-shaped strain distribution is observed and remains until the failure. The transition between these two different behaviors can be identified as the instant when the bond strength has been reached. After the debonding process has started, the strain gauges placed along the bonded length registered progressively increasing values of longitudinal strains while the shear force increased. Thus, a translation of the zone capable of stress transfer along the bonded length is observed with increasing of shear force during the debonding process.

In order to establish comparisons, Figure 10c shows the recorded strains from specimens with different concrete surface preparation methods. At the low level of applied load (10 kN), the strain profiles are very similar. However, in the SB specimen a steeper drop in the longitudinal strains was observed, which may indicate a higher initial stiffness. In the case of the highest load level concerning to the debonding process (25 kN), the influence of the concrete surface preparation method used on the interface behavior is clearer. At this same loading level, the strains recorded along the CFRP laminate of the SB specimen were significantly lower than those recorded in the GR specimen, which means a greater interface capacity in stress transfer between the CFRP and the concrete surface. Nevertheless, GR method mobilized longer bond regions in shear stress transfer. This would be expected since, due to the lower efficiency of GR method, it was necessary to mobilize a longer bond length to transfer the same amount of shear stress.

### 3.5. Effect of Concrete Surface Roughness on Bond Behavior—Analytical Approach

Once the influence of the concrete surface roughness on the EBR system performance was recognized, in this section the formulation proposed by the Italian guideline CNR (National Research Council) [1] for the prediction of maximum shear force of EBR FRP systems in concrete is updated/recalibrated, since the actual formula does not account for the effect of concrete surface roughness. However, this standard recognizes the importance of performing an effective surface preparation before gluing the laminate on the concrete surface. Additionally, this standard also refers that the method applied should be sandblasting and shall provide a roughness degree of at least 0.3 mm.

**Figure 11:**
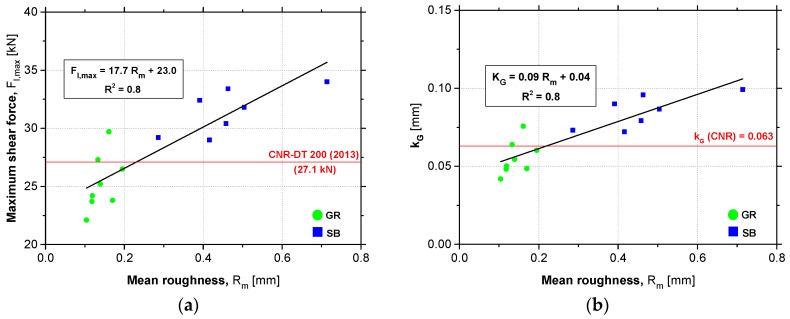
Relationship between the following parameters: (**a**) *F_l_*_,max_ vs. R_m_; (**b**) *k*_G_ vs. R_m_.

Figure 11a shows the relationship between the experimental maximum shear force obtained in the bond tests (*F_l_*_,max_) and the mean roughness coefficient obtained in the concrete surface roughness characterization (R_m_) through a linear tendency line (R^2^ = 0.8) established as follows: *F_l_*_,max_ = *m* × R_m_ + *b*, where *m* represents the effectiveness of increasing the roughness and *b* is the limit strength if a theoretical nil R_m_ value (perfectly smooth surface) could be obtained. In the formulation of the previous equation, only the results of the test series with bond length of 200 and 250 mm were included since these L_b_ values are not lower than the effective anchorage length (*l*_e_) calculated according to CNR [1] (Equation (4)). According to these predictions for the concrete test specimens used in this experimental work, the *l*_e_ value from which the increase of L_b_ no longer means the increase of bond strength is 200 mm. It is also important to refer that the specimen SB_Lb200_3 was not included in the analytical approach presented in this section, taking into account the corresponding observed failure mode. The predicted bond strength proposed by the Italian guideline is established by Equation (5). Applying this equation for that case, the maximum shear force predicted (*F*_max_) is 27.1 kN, as represented by the red line in Figure 11a. In Equations (4) and (5), *b*_f_, *t*_f_ and *E*_f_ are the width, thickness and Young’s modulus of the CFRP material, whereas Γ_Fm_ is the fracture energy. In turn, *γ*_Rd_ is a corrective factor with a value of 1.25 and *f*_b_ represents the mean bond strength obtained as a function of the parameter Γ_Fm_. The fracture energy (Γ_Fm_) is obtained by Equation (6) where *k*_b_ is a geometrical corrective factor obtained as a function of the ratio between the width of the CFRP laminate (*b*_f_) and the width of concrete specimen (*b*) (Equation (7)) and *k*_G_ is an additional experimental corrective factor which, according to CNR guideline [1], for the case of pre-cured FRP systems the average value is 0.063 mm. The parameters *f*_cm_ and *f*_ctm_ represent the average compressive and tensile strength of concrete, respectively.

(4) $$l_{e}=\max\left\{\frac{1}{\gamma_{\mathrm{Rd}}\cdot f_{b}}\sqrt{\frac{\pi^{2}\cdot E_{f}\cdot t_{f}\cdot \Gamma_{\mathrm{Fm}}}{2}},\;200\;\mathrm{mm}\right\},$$

(5) $$F_{\max}=b_{f}\cdot \sqrt{2\cdot E_{f}\cdot t_{f}\cdot \Gamma_{\mathrm{Fm}}},$$

(6) $$\Gamma_{\mathrm{Fm}}=k_{b}\cdot k_{G}\cdot \sqrt{f_{\mathrm{cm}}\cdot f_{\mathrm{ctm}}},$$

(7) $$k_{b}=\sqrt{\frac{2-b_{f}/b}{1+b_{f}/b}}\geq 1\;if\;b_{f}/b\geq 0.25.$$

Analyzing Figure 11a, it is remarkable that the formulation presented by the CNR [1] can be considered as a good prediction of bond strength when the concrete surface preparation method used has a limited efficiency. By comparing the prediction of *F*_max_ provided by the guideline with the experimental values of maximum shear force obtained in SB specimens, it is clear that improvements in the formulation are needed.

Therefore, in order to improve the analytical formulation taking into account the influence of the concrete surface roughness prior to the EBR system installation and allow a better prediction of the bond strength when a more effective surface preparation method is used, the following procedure was adopted: (i) using the Equation (5) and considering the value *F*_max_ equal to the maximum shear force obtained from experimental tests, the average experimental values of Γ_Fm_ were calculated; then, (ii) using the values of Γ_Fm_ inside Equation (6) and all the other known parameters, the experimental values of *k*_G_ were obtained. Figure 11b shows the relation between the experimental *k*_G_ values and the mean roughness coefficient obtained in the concrete surface roughness characterization (R_m_) through a linear tendency line (R^2^ = 0.8). In the same figure, the *k*_G_ value proposed by the Italian guideline is represented by a red line. As concluded by the accuracy of the maximum shear force prediction, it is also verified that the *k*_G_ mean value suggested by the CNR [1] can accurately predict the experimental values obtained when the concrete surface preparation method adopted does not provide high levels of roughness. The same observation cannot be drawn when a more effective surface preparation method is used. In the SB specimens, the *k*_G_ value obtained proved to be higher than that suggested by the formulation included in the CNR guideline.

To improve the *F*_max_ prediction (Equation (5)) maintaining the *k*_G_ value suggested by the guideline, a new parameter (*k*_R_—roughness corrective factor) was introduced in the calculation of the average interface fracture energy (Γ_Fm_), which is defined as a function of the mean roughness coefficient (R_m_), as demonstrated by Equations (8) and (9). From GR to SB, values of *k*_R_ ranged between 0.8 and 1.7, respectively. According to the obtained results, the CNR guideline [1] considers in its formulation (*k*_R_ = 1) a surface preparation method slightly more effective than the GR (*k*_R_ = 0.9, in average terms) and markedly less effective than the SB (*k*_R_ = 1.4, in average terms). In the absence of any information about the concrete surface roughness, it is recommended to use a value of *k*_R_ equal to 1, as suggested in the CNR standard.

**Figure 12:**
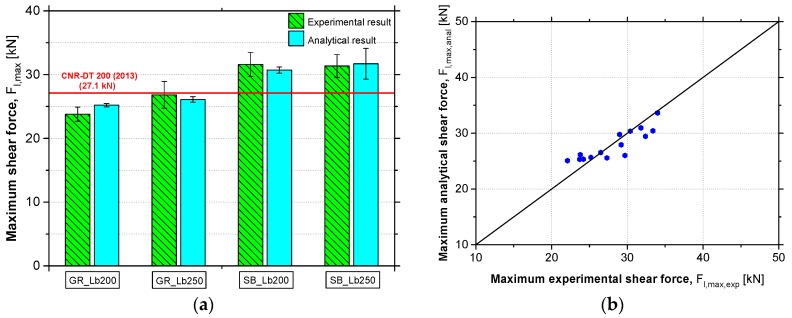
Comparison between the experimental results, the analytical results and the prediction provided by National Research Council [1] (**a**) and the accuracy of the proposed analytical model (**b**).

In order to assess the accuracy of the proposed analytical formulation, the average values of maximum shear force for each test series, obtained from the analytical model and from the experimental tests are compared with the prediction provided by the CNR guideline [1] in Figure 12a. The values of maximum shear force for each specimen, obtained from the analytical model and from the experimental tests are also compared in Figure 12b. From these figures, it is possible to verify the high accuracy of the proposed analytical model. The difference between analytical and experimental results was determined by Equation (10). Through the inclusion of the roughness corrective factor (*k*_R_) on existing CNR formulation for predicting the *F*_max_, the error values between the analytical formulation results and the experimental results were reduced from 11.8% to 4.4%.

(8) $$k_{R}=\frac{0.09R_{m}+0.04}{0.063}=1.4{\;R}_{m}+0.7,$$

(9) $$\Gamma_{\mathrm{Fm}}=k_{b}\cdot k_{G}\cdot k_{R}\sqrt{f_{\mathrm{cm}}\cdot f_{\mathrm{ctm}}},$$

(10) $$\%\;\mathrm{RPE}=\frac{\left|F_{l,\max,\exp}-F_{l,\max,\mathrm{anal}}\right|}{F_{l,\max,\exp}}\times 100.$$

## 4. Conclusions

This paper presented and analyzed the obtained results from an experimental program composed of 24 single-lap shear tests in prismatic concrete specimens with CFRP laminates applied according to the EBR technique. The aim of this work was to study the influence of some important parameters on the bond behavior between concrete and the CFRP laminate, namely: (i) the type of concrete surface preparation method used before the application of EBR system that provides different levels of surface roughness (GR—grinding and SB—sandblasting) and (ii) the bond length (150, 200 and 250 mm). For this purpose, the roughness of the distinct concrete surfaces was assessed by using a laser sensor in order to allow the identification of its influence on the bond strength. Additionally, the formulation proposed by the Italian guideline CNR [1] for the prediction of maximum shear force of EBR FRP systems in concrete was updated/recalibrated, since the actual formulation does not account for the effect of concrete surface roughness. Thus, the following main conclusions can be highlighted:

- Based on the measurements performed with the laser sensor, the results clearly showed that the SB method produced a higher level of concrete surface roughness than the GR. In terms of mean roughness coefficient (R_m_) and the root mean square (R_q_), the SB method yielded to an average increase of 250% and 150%, respectively, in relation to the surface preparation with GR;
- Different roughness levels, provided by the different preparation methods of the concrete surface, resulted in different levels of bond strength;
- Cohesive debonding in the concrete was the governed failure mode observed in all tests carried out, regardless of the surface preparation method used and bond length adopted. After debonding failure, a concrete layer attached to the epoxy adhesive and CFRP laminate was observed. In SB specimens, this concrete layer was considerably thicker than the layer observed in GR specimens, which reflects the better bond behavior provided by the SB method;
- The analysis of the *F_l_*-*s_l_* curves allowed to verify that the different preparation methods of the concrete surface did not significantly change the initial stiffness of the strengthening system. However, the debonding process started at higher shear force values in the case of SB specimens compared to the GR specimens. Thus, in turn, the increase of L_b_ value did not influence the *F_l_*-*s_l_* curves shape neither in the initial branch nor in the stiffness degradation phase and the debonding process;
- The use of the SB method instead of GR provided an increase of maximum shear force (*F_l_*_,max_) and total energy (*G*_f_), allowing a better use of the tensile strength capacity of the CFRP laminate;
- The use of higher L_b_ values provided an increase of maximum shear force (*F_l_*_,max_) and total energy (*G*_f_);
- Based on the obtained results from the experimental program, the influence of the surface roughness was included in the analytical formulation for predicting the maximum shear force proposed by CNR [1], through the inclusion of the roughness corrective factor (*k*_R_) defined as a function of the mean roughness coefficient (R_m_). The proposed analytical model proved to be able to accurately predict the bond strength of the EBR FRP system taking into account the roughness level of the concrete surface where the CFRP laminate is subsequently installed.

## Figures and Tables

**Table 1:** Properties of laser sensor SICK OD2—N50W10U0 and roughness measurement system.

Parameter	Value	Roughness Measurement System
Measuring range	40 to 60 mm	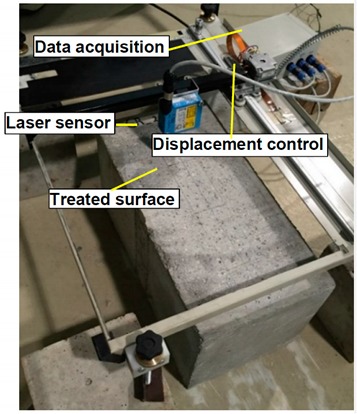
Resolution	5 μm	
Repeatability	15 μm	
Measuring frequency	2 kHz	
Light spot dimension (distance)	0.5 mm × 1 mm (50 mm)	

**Table 2:** Results from the roughness assessment (average values).

Series	Roughness Parameter [mm]	
	Mean Roughness Coefficient (Absolute Value), R_m_	Root Mean Square, R_q_
GR_Lb150	0.137 (21.8%)	0.260 (32.7%)
GR_Lb200	0.120 (10.3%)	0.181 (11.5%)
GR_Lb250	0.165 (13.4%)	0.329 (29.4%)
**Mean value**	0.141	0.256
SB_Lb150	0.533 (17.9%)	0.655 (21.8%)
SB_Lb200	0.460 (14.8%)	0.570 (9.4%)
SB_Lb250	0.491 (31.1%)	0.691 (32.7%)
**Mean value**	0.494	0.639

Notes: the values in parentheses are coefficients of variation (CoV).

**Table 3:** Material characterization (average values).

**Concrete**				
**Age of curing**	***f*_cm_** (MPa)		***E*_cm_** (GPa)	
28 days	33.4 (4.33%)		30.8 (2.84%)	
**CFRP**				
**Cross-section geometry** (mm^2^)	***f*_fu_** (MPa)	***E*_f_** (GPa)		***ε*_fu_** (%)
50 × 1.2	2222.4 (4.7%)	176.4 (2.0%)		1.2 (2.2%)
**Adhesive**				
**Type of adhesive**	***f*_a_** (MPa)	***E*_a_** (GPa)		***ε*_a_** (%)
S&P Resin 220 epoxy adhesive ^a^	22.0 (4.5%)	7.2 (3.7%)		0.36 (15.2%)

Notes: the values in parentheses are coefficients of variation (CoV); ^a^ Results collected from [21].

**Table 4:** Main results obtained from the single-lap shear tests (average values).

Series	*F_l_*_,max_ (kN)	*s_l_*_,max_ (mm)	*G*_f_ (kN·mm)	*ε*_fmax_ (%)	FM
GR_Lb150	23.8 (6.9%)	0.34 (11.7%)	5.08 (5.4%)	0.22	D [4]
GR_Lb200	23.8 (4.7%)	0.34 (18.0%)	5.37 (6.6%)	0.22	D [4]
GR_Lb250	26.8 (7.9%)	0.35 (19.2%)	5.89 (6.2%)	0.25	D [4]
SB_Lb150	27.2 (6.7%)	0.31 (12.5%)	5.79 (3.0%)	0.26	D [4]
SB_Lb200	30.2 (9.7%)	0.41 (8.2%)	5.92 (3.7%)	0.29	D [4]
SB_Lb250	31.3 (5.7%)	0.52 (24.0%)	6.07 (2.9%)	0.30	D [4]

Notes: the values in parentheses are coefficients of variation (CoV); D = cohesive debonding in the concrete; the values between brackets [ ] are the number of specimens with the specified failure mode.
